# Retraction: Application of an Isolated Word Speech Recognition System in the Field of Mental Health Consultation: Development and Usability Study

**DOI:** 10.2196/98661

**Published:** 2026-04-28

**Authors:** JMIR Publications Editorial Office

**Affiliations:** 1 JMIR Publications Toronto, ON

The JMIR Publications Editorial Office is retracting the article:

Application of an Isolated Word Speech Recognition System in the Field of Mental Health Consultation: Development and Usability Study [1]

This action is being taken due to identified concerns regarding potential manipulation of the submission process, the integrity of the peer review process, and the relevance of several references.

The concerns with this publication were identified during an investigation of a series of articles with related concerns. Author Weifeng Fu was contacted and offered an opportunity to comment on these findings and the proposed retraction but did not respond to any attempts at communication.
